# Supplementary material for: Strengthening Primary Health Care Through Implementation Research: Strategies for Reaching Zero-Dose Children in Low- and Middle-Income Countries’ Immunization Programs
Source: Vaccines (Basel). 2025 Oct 9;13(10):1040. doi: 10.3390/vaccines13101040 (PMC12568197; doi:10.3390/vaccines13101040)
Supplement: Supplementary file 1 [file vaccines-13-01040-s001.zip › S1 Summary 36 immunization related IR projects included 1.pdf]

A summary of all the immunization related IR projects included

Author and title	Study design and methods	Country	Intervention focus areas
Abakar et al.[1], Evaluation of the feasibility and sustainability of the joint human and animal vaccination and its integration to the public health system in the Danamadji health district, Chad	***Mixed-methods study*** incorporating semi-structured interviews, FGDs, and budget impact analysis.	Chad	Integrated approaches through the One Health joint vaccination initiative
Ahmed et al. (Unicef report) [2, 3], E-Vaccs: Assessment of barriers and enablers using the Consolidated Framework for Implementation Research in Punjab	***Qualitative study*** using key informant interviews (KIIs).	Pakistan	Innovation and technology focusing on use of technology for performance management of tracking coverage
Akwataghibe et al.[4], Using participatory action research to improve immunization utilization in areas with pockets of unimmunized children in Nigeria	***Mixed methods, pre/post-intervention design***, combining household surveys with caregivers, semi structured interviews with immunization stakeholders, and FGD with community members.	Nigeria	Research and evidence generation through participatory action research for community sensitization and demand generation in immunization
Altaf et al.[5], Visibility and Analytics Network (VAN) approach to improve immunization supply chain and management performance system in Pakistan	***A mixed-methods approach***, combining quantitative assessments of EPI storage facilities with interviews of data entry personnel using a pre-tested tool on immunization supply chain management, alongside in-depth interviews with senior VAN project managers.	Pakistan	Supply chain and logistics on the immunization supply chain management
Amare et al.[6], Vaccine Safety Practices and Its Implementation Barriers in Northwest Ethiopia	***A qualitative approach***, using face-to-face IDI with key informants and direct observations of immunization sessions.	Ethiopia	Vaccine hesitancy and acceptance touching on vaccine safety practices
Asrade et al.[7], The Burden of Internal Conflict on Expanded Programs on Immunization in Northwest Ethiopia	***A mixed-methods approach*** was used, combining record reviews with a phenomenological design and in-depth interviews	Ethiopia	Immunisation in emergency and conflict settings
Bayih et al.[8], The Implementation of Social and Behaviour Change Communication Intervention to Improve Immunization Demand	***A phenomenological qualitative study*** was conducted through in-depth interviews with fifteen key informants using a piloted semi-structured interview guide.	Ethiopia	Demand generation and community engagement through social and behaviour change

			communication (SBCC) for immunization
Dadari et al.[9], Pro-equity immunization and health systems strengthening strategies in select Gavi-supported countries	***A review*** of country reports and consultations with UNICEF and Gavi staff examined pro-equity immunization activities implemented through HSS strategies at national and subnational levels.	Afghanistan, Central African Republic (CAR), Chad, Ethiopia, India, Kenya, Kyrgyzstan, Madagascar, Myanmar, Nigeria, Pakistan, Uganda	Cross-cutting areas include demand generation and community engagement (dominant); governance and workforce; integrated approaches; supply chain and logistics; and data systems and decision support, all contributing to equity and health system strengthening.
Endehabtu et al.[10], How to Optimize Immunization Supply Management at Different Levels of the Health System in Oromia Region: An Implementation Science Research	A ***phenomenological qualitative approach***, using interview guides with 27 key informants across health posts, health centres, woreda health offices, a regional hub, and the Ethiopian Pharmaceutical Supply Agency	Ethiopia	Supply chain and logistics on the component of immunization supply chain management
Etokidem et al.[11], Potential barriers to and facilitators of civil society organization engagement in increasing immunization coverage in Odukpani Local Government Area of Cross River State, Nigeria	***Qualitative*** methods, including 22 FGDs, 3 IDIs, and 26 KIIs.	Nigeria	Demand generation and community engagement through civil society engagement in immunization
Gedlu et al.[12], Implementation Fidelity and Challenges of Optimal Cold Chain Management in Assossa District Health System, Benshangul-Gumuz Region, Western Ethiopia	A ***qualitative approach*** using observational methods and interviews with 12 key informants.	Ethiopia	Supply chain and logistics on the component of cold chain management
Gelagay et al.[13], Full Immunization Coverage and Its Determinants among	***A community-based cross-sectional study*** using an interviewer-administered questionnaire.	Ethiopia	Data systems and decision support to enhance

Children Aged 12-23 Months in Wogera District, Northwest Ethiopia			immunization coverage and determinants
Gelagay et al.[14], Women's Independent Household Decision Making Power and Its Influence on Their Autonomy in Relation to Child Vaccinations	***A community-based study*** employing a ***concurrent mixed-methods design***: cross-sectional for quantitative and phenomenological for qualitative data	Ethiopia	Vaccine hesitancy and acceptance by supporting women's decision-making and child vaccinations
Haq and Rana [15], Synergized immunization programming: Pakistan's road to polio eradication	***A review and descriptive analysis***	Pakistan	Integrated approaches by polio eradication through synergised immunization – Zero-dose
Haq et al.[16], The synergy between Expanded Program on Immunization and Polio Eradication Initiative in Pakistan: a policy and program exploration	A ***national narrative study*** using semi-structured interviews with program managers and policymakers and FGDs with vaccinators and CBV workers.	Pakistan	Integrated approaches by polio eradication through synergised immunization – Zero-dose
Haq et al.[17], System within systems: challenges and opportunities for the Expanded Programme on Immunisation in Pakistan	a ***qualitative case study*** using semi-structured in-depth interviews with 34 purposively selected key informants	Pakistan	Immunization planning and delivery by addressing immunization challenges
Kefiyalew et al.[18], Assessment of immunization data management practices, facilitators, and barriers to immunization data quality in the health facilities of Tach Gayint district, Northwest Ethiopia	A ***mixed-method study design*** was employed, using document review of 18 health facilities for quantitative data and 26 key informant interviews with immunization experts for qualitative insights.	Ethiopia	Data systems and decision support for enhancing the immunization data quality and management
Kefiyalew et al.[19], Assessment of barriers to the implementation of community-based data verification and immunization data discrepancies between health facilities and the community in Tach Gayint district, Northwest Ethiopia	A ***mixed-methods design***, combining a phenomenological approach to explore barriers to Community-Based Data Validation (CBDV) with a facility- and community-based cross-sectional survey assessing discrepancies between immunization data reported by health facilities and communities.	Ethiopia	Data systems and decision support through immunization data verification
Kerebih et al.[20], How to optimize health facilities and community linkage in order to	A ***phenomenological explanatory study*** using structured interviews with 46 key informants.	Ethiopia	Demand generation and community engagement through

enhance immunization service: the case of West Amhara Region, Ethiopia			health facility and community linkage for immunization
Khan et al. (UNICEF report) [2, 3], Addressing EPI vaccination demand through mHealth in Quetta City, Balochistan: A feasibility study	A mixed-methods study – using baseline data from 1,600 parents across 75 BHUs Balochistan, and an endline survey with 1,203 participants; three KIIs (two Lady Health Supervisors and one WHO routine immunization officer) and three FGDs.	Pakistan	Innovation and technology through the use of technology and AI/ mHealth to increase demand
Khan et al.[21], Developing a three-dimensional narrative to counter polio vaccine refusal in Charsadda	This study adopted ***a relativist ontological and interpretive epistemological stance***, using ***a case study design*** based on 43 in-depth interviews.	Pakistan	Vaccine hesitancy and acceptance
Madebo et al.[22], Immunization Data Quality and Factors Influencing Data Generation, Handling and Use in Wogera District, Northern Ethiopia	***A mixed-methods design*** was used, combining immunization record reviews from 41 primary health facilities with in-depth interviews of 20 health workers.	Ethiopia	Data systems and decision support by enhancing immunization data quality
Mafigiri et al.[23], A qualitative study of the development and utilization of health facility-based immunization micro plans in Uganda	***A comparative qualitative study*** conducted in two districts used multilevel observations, micro plan and meeting records review, and 57 interviews with ministry and frontline health workers.	Uganda	Immunization planning and delivery through immunization microplanning
Malik et al.[24], Social mobilization campaign to tackle immunization hesitancy in Sargodha and Khushab districts of Pakistan	***A mixed-methods approach*** was employed, combining quantitative data from 329 community health workers with qualitative insights from key informant interviews with senior EPI officials and focus group discussions with parents.	Pakistan	Demand generation and community engagement
Mancuso et al.[25], Cross-cutting lessons from the Decision-Maker Led Implementation Research initiative	This study employed ***qualitative methods***, including document review and in-depth interviews with decision-makers and researchers involved in the initiative.	Chad, Ethiopia, India, Nigeria, Pakistan, and Uganda	Cross-cutting areas include research and evidence generation, immunization planning and delivery, governance and workforce, and data systems and decision support, all underpinned by

			decision-maker-led implementation research.
Mekonnen et al.[26], Strategies to Revitalize Immunization Service Provision in Urban Settings of Ethiopia	***A qualitative phenomenological study*** was conducted using data from 35 key informant interviews and 9 in-depth interviews across health system and community levels.	Ethiopia	Immunization planning and delivery through urban immunization strategies
Nair et al.[27], Social media, vaccine hesitancy and trust deficit in immunization programs	***A qualitative study*** involving in-depth interviews and focus group discussions with parents/caregivers, healthcare providers, public health staff, alternative medicine practitioners, frontline workers, and teachers.	India	Vaccine hesitancy and acceptance by enhancing trust
Naveed et al.[28], Understanding the accountability issues of the immunization workforce for the Expanded Program on Immunization (EPI) in Balochistan	A ***qualitative exploratory study*** was conducted using semi-structured, open-ended interviews with provincial and district EPI staff and District Health Officers (DHOs).	Pakistan	Governance and workforce through EPI workforce accountability – Zero-dose
Qayyum et al.[29], Addressing community barriers to immunization in Rajanpur district, Pakistan: an implementation research	An ***exploratory qualitative study*** comprising 24 in-depth interviews and 7 focus group discussions with community members, caregivers, and healthcare providers.	Pakistan	Demand generation and community engagement
Sahito et al.[30], Covering the last mile for vaccination: Feasibility and acceptability of traditional birth attendant-based referral system in hard-to-reach areas in rural Pakistan	A ***mixed-method design*** was used, combining a quasi-experimental pre- and post-intervention study with a control group, alongside qualitative inquiry.	Pakistan	Immunization planning and delivery through vaccination outreach
Sana et al (Unicef report) [2, 3], Strengthening supportive supervision: A case study of the Expanded Programme on Immunization in Sindh	An ***exploratory case study*** was conducted using 11 KIIs, 5 FGD, EPI checklist observations, and a review of national EPI policy and WHO's Mid-Level Manager Training Module 4.	Pakistan	Governance and workforce through enhancing supportive supervision
Shahabuddin et al.[31], An embedded implementation research initiative to tackle	A ***mixed-methods study*** using data collected from IR team members through an online survey and	Pakistan	Immunization planning and delivery by enhancing immunization service delivery

service delivery bottlenecks in the expanded programme on immunisation in Pakistan	follow-up in-depth interviews, both by phone and in person.		
Shahabuddin et al.[32], Carrying out embedded implementation research in humanitarian settings: A qualitative study in Cox's Bazar, Bangladesh	A ***qualitative study*** conducted in two phases, involving in-depth interviews with representatives from non-governmental organizations (NGOs), United Nations (UN) agencies, and the Government of Bangladesh.	Bangladesh	Embedded research in humanitarian setting enhancing care in conflict settings
Tilahun et al.[33], Using health data for decision-making at each level of the health system to achieve universal health coverage	***Mixed-methods implementation research*** combining document reviews with interviews of 21 key informants (community representatives, data producers, users, and decision-makers at local to regional levels).	Ethiopia	Data systems and decision support through use of health data for decision-making
Yazdani et al.[34], Unveiling and addressing implementation barriers to routine immunization in the peri-urban slums of Karachi	Used a ***mixed-methods design*** combining a baseline cross-sectional survey with in-depth interviews.	Pakistan	Immunization planning and delivery addressing routine immunization barriers
Zewde et al.[35], The influence of linkages, feedback mechanisms, and caregiver mobility on immunization follow-up visits	A ***qualitative approach*** was used, involving 30 in-depth interviews with caregivers, 26 key informant interviews, and five focus group discussions with health officials and decision-makers.	Ethiopia	Immunization planning and delivery enhancing immunization follow-up visits

References

1. Abakar, M.F., et al., *Evaluation of the feasibility and sustainability of the joint human and animal vaccination and its integration to the public health system in the Danamadji health district, Chad.* Health Research Policy and Systems, 2021. **19**(2): p. 44.
2. UNICEF. *Implementation research: Country projects*. 2025 [cited 2025 24 January]; Available from: https://irds.quo-staging.com/country-projects/#reporting.
3. UNICEF, *Pakistan Implementation Research for Immunization – A compilation of project objectives, results and recommendations*. 2018, United Nations Children's Fund (UNICEF),

Alliance for Health Policy and Systems Research,

Gavi the Vaccine Alliance,.

4. Akwataghibe, N.N., et al., *Using participatory action research to improve immunization utilization in areas with pockets of unimmunized children in Nigeria.* Health Research Policy and Systems, 2021. **19**(2): p. 88.
5. Altaf, A., et al., *Visibility and Analytics Network (VAN) approach to improve immunization supply chain and management performance system in Pakistan.* Journal of Global Health, 2021. **11**.
6. Amare, G., et al., *Vaccine safety practices and its implementation barriers in Northwest Ethiopia: A qualitative study.* Ethiopian Journal of Health Development, 2021. **35**(3).
7. Asrade, G., et al., *The burden of internal conflict on expanded programs on immunization in northwest Ethiopia: implementation science study.* Ethiopian Journal of Health Development, 2021. **35**(3).
8. Bayih, G., et al., *The implementation of social and behavior change communication intervention to improve immunization demand: a qualitative study in Awabel district, Northwest Ethiopia.* Ethiopian Journal of Health Development, 2021. **35**(3).
9. Dadari, I., et al., *Pro-equity immunization and health systems strengthening strategies in select Gavi-supported countries.* Vaccine, 2021. **39**(17): p. 2434-2444.
10. Endehabtu, B.F., et al., *How to optimize Immunization Supply Management at different levels of the health system in Oromia Region? An implementation science research.* Ethiopian Journal of Health Development, 2021. **35**(3).
11. Etokidem, A., et al., *Potential barriers to and facilitators of civil society organization engagement in increasing immunization coverage in Odukpani Local Government Area of Cross River State, Nigeria: an implementation research.* Health Research Policy and Systems, 2021. **19**(2): p. 46.
12. Gedlu, T., et al., *Implementation fidelity and challenges of optimal cold chain management in Assossa district health system, Benshangul-Gumuz region, Western Ethiopia.* Ethiopian Journal of Health Development, 2021. **35**(3).
13. Gelagay, A.A., et al., *Full immunization coverage and its determinants among children aged 12-23 months in Wogera district, Northwest Ethiopia.* Ethiopian Journal of Health Development, 2021. **35**(3).
14. Gelagay, A.A., et al., *Women's Independent Household Decision Making Power and its influence on their Autonomy in relation to Child Vaccinations: a mixed-method study among Women of Reproductive Age in Northwest Ethiopia.* Ethiopian Journal of Health Development, 2021. **35**(3).
15. Ul Haq, Z. and M.S. Rana, *Synergized immunization programming: Pakistan's road to polio eradication.* Journal of Global Health, 2021. **11**.

16. Haq, Z., et al., *The synergy between Expanded Program on Immunization and Polio Eradication Initiative in Pakistan: a policy and program exploration.* Journal of Global Health Reports, 2021. **5**: p. e2021081.
17. Haq, Z., et al., *System within systems: challenges and opportunities for the Expanded Programme on Immunisation in Pakistan.* Health Research Policy and Systems, 2019. **17**(1): p. 51.
18. Kefiyalew, B., et al., *Assessment of immunization data management practices, facilitators, and barriers to immunization data quality in the health facilities of Tach Gayint district, Northwest Ethiopia.* Ethiopian Journal of Health Development, 2021. **35**(3).
19. Kefiyalew, B., et al., *Assessment of barriers to the implementation of community-based data verification and immunization data discrepancies between health facilities and the community in Tach Gayint district, Northwest Ethiopia.* Ethiopian Journal of Health Development, 2021. **35**(3).
20. Kerebih, M., et al., *How to optimize health facilities and community linkage in order to enhance immunization service? The case of West Amhara Region, Ethiopia.* Ethiopian Journal of Health Development, 2021. **35**(3).
21. Khan, S.A., et al., *Developing a three-dimensional narrative to counter polio vaccine refusal in Charsadda.* Journal of Global Health, 2020. **10**(2).
22. Madebo, T.H., et al., *Immunization data quality and factors influencing data generation, handling and use in Wogera District, Northern Ethiopia, 2020.* Ethiopian Journal of Health Development, 2021. **35**(3).
23. Mafigiri, D.K., et al., *A qualitative study of the development and utilization of health facility-based immunization microplans in Uganda.* Health Research Policy and Systems, 2021. **19**(2): p. 52.
24. Malik, M.N., M.S. Awan, and T. Saleem, *Social mobilization campaign to tackle immunization hesitancy in Sargodha and Khushab districts of Pakistan.* Journal of Global Health, 2020. **10**(2).
25. Mancuso, A., et al., *Cross-cutting lessons from the Decision-Maker Led Implementation Research initiative.* Health Research Policy and Systems, 2021. **19**(2): p. 83.
26. Mekonnen, Z.A., et al., *Strategies to revitalize immunization service provision in urban settings of Ethiopia.* Ethiopian Journal of Health Development, 2021. **35**(3).
27. Nair, A.T., et al., *Social media, vaccine hesitancy and trust deficit in immunization programs: a qualitative enquiry in Malappuram District of Kerala, India.* Health Research Policy and Systems, 2021. **19**(2): p. 56.
28. Naveed, Z., et al., *Understanding the accountability issues of the immunization workforce for the Expanded Program on Immunization (EPI) in Balochistan: An exploratory study.* Journal of Global Health, 2021. **11**.
29. Qayyum, K., et al., *Addressing community barriers to immunization in Rajanpur district, Pakistan: an implementation research.* Journal of Global Health Reports, 2021. **5**: p. e2021088.
30. Sahito, A., S. Ahmed, and Z. Fatmi, *Covering the last mile for vaccination: Feasibility and acceptability of traditional birth attendant-based referral system in hard-to-reach areas in rural Pakistan.* Journal of Global Health, 2020. **10**(2).
31. Shahabuddin, A., et al., *An embedded implementation research initiative to tackle service delivery bottlenecks in the expanded programme on immunisation in Pakistan: Overview and reflections.* Journal of Global Health, 2021. **11**.
32. Shahabuddin, A., et al., *Carrying out embedded implementation research in humanitarian settings: A qualitative study in Cox's Bazar, Bangladesh.* PLoS Medicine, 2020. **17**(7): p. e1003148.

33. Tilahun, B., et al., *Using health data for decision-making at each level of the health system to achieve universal health coverage in Ethiopia: the case of an immunization programme in a low-resource setting.* Health Research Policy and Systems, 2021. **19**(2): p. 48.
34. Yazdani, A.T., et al., *Unveiling and addressing implementation barriers to routine immunization in the peri-urban slums of Karachi, Pakistan: a mixed-methods study.* Health Research Policy and Systems, 2021. **19**(2): p. 55.
35. Zewde, T., et al., *The influence of linkages, feedback mechanisms, and caregiver mobility on immunization follow-up visits in Lideta sub-city of Addis Ababa, Ethiopia: a qualitative study.* Health Research Policy and Systems, 2021. **19**(2): p. 69.
